# When Being Bad Feels Good: A Systematic Review of the Relationship Between Positive Emotion and Antisocial Behavior in Children and Adolescents

**DOI:** 10.1007/s10567-024-00493-4

**Published:** 2024-07-03

**Authors:** Jessica Moore, Lok Yee Chloe Tam, Jennifer L. Allen

**Affiliations:** Department of Psychology, University of Bath, 10 West, Claverton Down, Bath, BA2 7AY UK

**Keywords:** Antisocial behavior, Aggression, Positive emotion, Children, Adolescents, Happy victimizer

## Abstract

Antisocial behavior in childhood and adolescence is associated with poor family and peer relationships, and a higher risk of mental and physical health problems in adulthood, as well as criminality. Emotions play a central role in children’s moral development, but most research has focused on negative emotions (e.g., shame and guilt), in relation to childhood antisocial behavior. Research in adult populations indicates that positive emotions experienced in anticipation of, during, and after antisocial acts may play an important role in the development and maintenance of antisocial behavior. Consequently, this systematic review aimed to investigate the relationship between positive emotion and antisocial behavior in children and adolescents. A systematic search in five databases was conducted, yielding 52 studies that used different methodological approaches, samples, designs and methods to examine this association. Results provide support for a positive relationship between positive emotion and antisocial behavior across community, forensic and clinical samples. This link appeared to be stronger for younger children, boys, and for children high in social dominance, callous-unemotional or sensation-seeking traits. Results suggested that positive affect may act in concert with negative emotion, cognitive, personality and motivational processes, as well as peer influences to determine the initiation and maintenance of antisocial behavior. This review presents directions for future research and discusses the implications of findings for prevention and intervention programs for youth with antisocial behavior.

**Supplementary Information:**

The online version contains supplementary material available at 10.1007/s10567-024-00493-4.

## Introduction

### Antisocial Behavior in Childhood and Adolescence

Antisocial behavior (AB) is broadly defined as actions or attitudes that violate societal norms and/or the personal and property rights of others, such as stealing, vandalism, aggression, and bullying (Farrington, 2005). When these behaviors are severe, persistent and accompanied by significant impairment in family, peer, educational, or occupational settings, a diagnosis of one of two forms of disruptive behavior disorders may be warranted: Oppositional Defiant Disorder (ODD) and Conduct Disorder (CD; Allen et al., 2020). These disorders are common, with the prevalence of ODD estimated at 3.3% for children aged 5–18 years in community samples (Canino et al., 2010) and from 28 to 65% in clinical samples (Boylan et al., 2007). CD is estimated to have a lifetime prevalence of 9.5% (12% among males, and 7% among females), with a median age of onset of 11.6 years (Nock et al., 2006). These disorders tend to be more prevalent in boys than girls, although this gender imbalance diminishes in adolescence (Hawes et al., 2023). AB in childhood is associated with poor family and peer relationships, truancy, early school leaving, social alienation and crime involvement (Moffitt, 2018). It is the most common reason for referrals to child psychiatric services (Pikard et al., 2018), and predicts later AB, as well as substance abuse, depression, and physical health problems (Odgers et al., 2007). Consequently, researchers have sought to understand factors influencing the onset and persistence of AB in children, including the role of emotions, to inform prevention and intervention work (Frick & Viding, 2009).

### The Role of Positive Emotion in Antisocial Behavior

Emotions, also termed feelings or affect, refer to a subjective, valenced experience that can be pleasant (positive) or unpleasant (negative) or both simultaneously (Chester, 2017). Chester described arousal as an orthogonal feature to valence, with more serious forms of AB typically viewed as arising from negative emotions experienced at higher levels of arousal such as anger/frustration, fear, and pain. More recent research has shifted its focus to positive emotions as both causes and correlates of AB (Chester & DeWall, 2017; Chester et al., 2019). It has long been known that people of all ages enjoy engaging in AB and aggression, albeit generally in mild, socially acceptable forms, evidenced by rough-and-tumble play, games that involve deceit to win, and sports that involve physical aggression. Similar to negative emotions, positive emotions experienced at moderate to high levels of arousal (e.g., joy, excitement) are more commonly associated with AB than low levels of arousal (e.g., calm, relaxation). Positive affect as a motivation for AB has been associated with mild to severe forms of AB in adults, including reactive aggression in response to threat/provocation or a perceived threat/provocation, and proactive aggression that is planned and intentional such as bullying and online ‘trolling’ (Chester, 2017).

### Theories of Positive Emotion and Antisocial Behavior in Adults

The idea that positive affect may drive antisocial and aggressive behavior is not new. Evolutionary theory describes humans as predatory organisms with motivational brain circuitry tied to predation (e.g., hunting), with positive affect selected for in the larger population (Chester, 2017). The pleasure derived from aggression has been proposed to then extend beyond hunting to aggressive acts that promote reproduction as well as survival (Griskevicius et al., 2009). Early theories viewed AB as a form of relief from negative affect. For example, the Freudian concept of ‘catharsis’ views aggression as a release from the long-term build-up of negative emotions, resulting in pleasant relief (Bushman, 2002). Contemporary adult theories have focused on emotion regulation and individual beliefs about the role of positive affect, either alone or in combination with negative affect, in relation to antisocial acts (Chester, 2017; DeWall et al., 2011). Positive emotion is viewed as forming a positive feedback loop, with the expectation that the antisocial act will lead to positive affect, increasing the likelihood of aggression, with this experience of positive affect during or following the act increasing the likelihood that the individual will repeat this behavior in future (Chester & DeWall, 2017; Gollwitzer & Bushman, 2012). In this model, aggression is viewed as a dynamic interaction between the negative affect that an individual currently feels and the positive affect that he or she expects to feel during the aggressive act. This idea of a positive feedback loop fits with the General Aggression Model (DeWall et al., 2011), which argues that aggression can be attributed to the interplay between individual characteristics (e.g., gender, personality), the situation, internal states (i.e., cognition, arousal, and affect), and the outcomes of appraisal and decision-making processes.

The Quadripartite Violence Typology (QVT) framework also highlights the role of positive emotion in understanding motivations for aggression (Howard, 2011). According to the QVT, an act of violence may be impulsive or premeditated, and these acts may be motivated by positive emotion (i.e., appetitive aggression) or negative emotion (aversive aggression). This 2 × 2 typology proposes four types of violence, each associated with the achievement of a particular goal: to elicit or improve positive mood (i.e., excitement in appetitive/impulsive violence), to gain material goods or social dominance (i.e., greed in appetitive/premeditated violence), or to reduce negative affect through the removal of threat (i.e., self-defence in aversive/impulsive violence) or retribution (i.e., revenge in aversive/premeditated violence). While the QVT retains the traditional distinction between impulsive and premeditated aggression, this model also incorporates the distinction between reactive and proactive aggression, thereby providing a rich representation of the variety of motivations that drive violent behavior.

QVT also incorporates aspects of reversal theory (Apter, 2007), which posits that negative emotions such as anger can be experienced as positive, particularly when a ‘protective frame’ such as physical or emotional detachment from the situation or victim is present (e.g., film or TV character). Anger may be appraised as a positive affective experience in the context of impulsive acts (‘thrill-seeking anger’) or controlled acts (‘coercive anger’), and to states of arousal-seeking (pleasant excitement) rather than arousal avoidance (unpleasant fear or anxiety). Similarly, the ‘fear enjoyment hypothesis’ posits that individuals high in psychopathic traits appraise their emotional experience in relation to fear-inducing stimuli or situations as positive, and thus these individuals are motivated to pursue such stimuli or situations despite the risk of harm to the self or others (Hosker-Field, Gauthier, & Book, 2016). In the QVT model, being in an arousal-seeking state is not enough to determine the valence of affect, it merely indicates that the individual desires excitement, and if not experienced, this may lead to negative emotions such as boredom or frustration. The valence of affect is attributed to the combination of the individual’s motivational state, the protective frame (appraisal of the level of safety or threat carried by the arousal state and the environment), and the environment itself.

More recent theories incorporate the role of motivation and attitudes towards emotions in explaining the link between positive affect and aggression, acknowledging the importance of emotion goals, or what people *want* to feel in driving behavior (Harmon-Jones et al., 2011; Millgram, Huppert, & Tamir, 2020). Emotion goals shape whether, how and when people regulate emotions (Tamir & Gutentag, 2017). Individuals who have a preference for positive emotion are more likely to select into situations likely to elicit this preferred emotional state, and are less motivated to down-regulate positive affect in the context of aggression. As such, the aggression of some individuals may reflect motivation rather than a lack of capacity to regulate emotions. Research on emotion goals and attitudes has also examined how these factors relate to individual differences, particularly personality traits. Antisocial acts motivated by positive affect (e.g., excitement-seeking) and the appraisal of ‘negative’ emotions as positive appear to be more likely when the individual is a younger male, under the influence of alcohol or other substances, in the presence of deviant peers, and/or when elevated levels of psychopathic traits, sensation-seeking, dominance, trait anger and sadism are present (e.g., Garofalo & Spantidaki Kyriazi, 2024; Howard, 2011; Hosker-Field et al., 2016; Spantidaki et al., 2023, 2024). This strong focus on personality traits is unsurprising given that most models of personality include emotional reactivity and regulation as a core dimension (Mervielde & Asendorpf, 2014). Indeed, impulsivity and sensation-seeking traits have positive affect as a motivational force embedded within their definition. Similarly, individuals high in dominance coupled with hostility/antagonism (e.g., taking resources from others, physical or relational aggression) derive pleasure from gaining power over others via antisocial means (Hawley, 2007), while sadism is defined as an persistent pattern of cruel and degrading behavior that results in distress for the victim and pleasure for the actor (O’Meara et al., 2011).

### Emotions and Antisocial Behavior in Children and Adolescents

Emotions play a central role in children’s moral development and therefore in the onset and persistence of AB (Eisenberg et al., 1987). Similar to research on adults, most research on child and adolescent AB has focussed on negative emotions, notably self-referential emotions such as guilt, shame, and sympathy. Research exploring positive emotion as a cause or correlate of AB in children is chiefly derived from three different areas of research: (1) moral emotion attributions such as the ‘happy victimiser’ phenomenon, (2) the emotional experiences of child bullies, and (3) peer influences. These different areas stem from different disciplines, and therefore often take different theoretical perspectives and favour different methodological approaches to investigate the potential link between positive emotion and AB. Thus the current review aims to describe and integrate theory and evidence from these different sources, identify gaps in knowledge, guide future research, and inform intervention.

### Positive Moral Emotion Attributions and Antisocial Behavior in Children and Adolescents

Moral emotion attributions are emotions that are attributed to morally relevant behavior, which can be positive or negative depending on the type of action (e.g., pride over prosocial actions, or guilt over a moral transgression; Malti & Krettenauer, 2013). At six or seven years of age, children typically begin to attribute negative emotions to moral transgressors (e.g., sadness, shame, or guilt), indicating that they understand the emotional outcomes of the sociomoral event for the transgressor (Krettenauer et al., 2008). Consequently, the emotions attributed to past events and behavior may help children to anticipate future outcomes, make appropriate moral judgements, adjust their moral behaviors accordingly and to develop empathy and perspective-taking skills (Helwig, 2007; Malti & Krettenauer, 2013). This emotional understanding also plays a role in the development of conscience, which acts as an internal moral compass to guide child behavior (Thompson, 2013). There is strong evidence for the importance of moral emotion attributions, with a meta-analysis including 42 studies with over 8000 participants aged 4–20 years showing significant relationships between these attributions and AB (Malti & Krettenauer, 2013).

While evidence suggests that children around the age of four or five years old can understand acts of victimisation as morally wrong from a cognitive point of view, they do not consistently attribute negative moral emotions (e.g., guilt, sadness, or remorse) following moral transgressions (see Arsenio et al., 2006, for a review). Interestingly, children at this age tend to report that transgressors will experience positive emotion, or the absence of negative emotion following a transgression, seemingly because they have satisfied their own interests. This phenomenon of attributing positive emotions to transgressors despite recognising that a moral rule was violated has been termed the ‘happy victimiser’ effect (Arsenio & Lover, 1995). For example, Nunner-Winkler and Sodian (1988) presented children aged four, six and eight years old with moral conflicts in which the character is tempted to transgress to satisfy a personal desire. Children’s understanding of the moral rules was explored, and when the character transgressed, children were asked to attribute an emotion to the wrongdoer. By the age of four, 98% of children knew that stealing was wrong, yet 80% of these children expected the wrongdoer to ‘feel good’ because the transgressor satisfied their own desires. Although the ‘happy victimiser’ phenomenon is mostly observed in pre-school aged children and is known to decrease with age (Nunner-Winkler, 1998), this effect has also been observed in later childhood and adolescence (Kunimatsu et al., 2012; Malti et al., 2013).

Different explanations have been proposed to explain this phenomenon. Nunner-Winkler (2007) proposed a theory of moral motivation, suggesting that the happy victimizer effect occurs because young children’s cognitive abilities have not yet fully developed to allow them to regulate their moral motivations (i.e., the willingness to give priority to moral concerns over tempting non-moral values). Their longitudinal findings in a community sample of preschool children (N = 213) assessed at 4, 6, 8, 17 and 22 years suggested that the strength of moral motivation (measured using a rating procedure based on the participant’s hypothetical action decisions and emotional reactions) increases with age. This motivational theory suggests that while young children understand moral rules, they do not experience these rules as personally binding and therefore transgressions do not lead to negative moral emotions such as remorse, and instead lead to positive or mixed emotions.

Other researchers have criticised this view for not considering the role of cognition in the happy victimiser phenomenon, arguing that cognition and motivation are not distinct processes, as children must be able to consider and predict the victimiser’s feelings which requires a cognitive appraisal of the situation (Krettenauer et al., 2008; Minnameier, 2012). As children’s cognitive abilities develop, they become increasingly able to understand that different events have different emotional outcomes (Arsenio et al., 2006). However, a meta-analysis by Malti and Krettenauer (2013) indicated that age did not moderate the relationship between moral emotion attributions and antisocial behavior, therefore they suggested that moral emotion attributions likely reflect individual differences in morally relevant behavioral dispositions, rather than age-related deficits in cognitive ability or moral motivation. In line with this view, Krettenauer et al. (2013) found that low conscientiousness and low agreeableness predicted the development of moral emotion attributions from childhood to middle adolescence and AB in adulthood. Consequently, child individual differences such as temperament or personality traits may have an important influence on the relationship between positive emotion and AB.

### Bullying, Positive Emotions and Antisocial Behavior in Children and Adolescents

Bullying is common in childhood and adolescence, and has negative consequences for bullies, victims, and bully-victims (Armitage, 2021). Bullying is characterised by repeated victimisation and encompasses a wide range of types, frequencies, and aggression levels, ranging from teasing and name calling to physical, verbal, and social abuse. Some children who bully appear to do so, at least in part because they experience pleasure in hurting others (Pfattheicher et al., 2023). Indeed, in an experimental study of 170 children, 23% were ‘amused’ by bullying scenarios (Boulton & Flemington, 1996). Another study exploring bullying using a peer-nomination procedure in a community sample of 573 children, identified six participant roles that children may have in the bullying process (Salmivalli et al., 1996). Some children join in with the bullying (assistants), some defend the victim (defenders), some do nothing (outsiders), but some laugh (a behavioral indicator of positive emotion) and further incite the bully while the bullying is occurring (reinforcers). These results suggest that positive emotion may play a role in the initiation and persistence of bullying for bullies and reinforcers of bullying.

Similar findings have been reported in research on cyberbullying in children. One study on middle school and high school students (*N* = 2186) found that participants bullied others online because it made them feel funny, popular, and powerful (Mishna et al., 2010). Fun-seeking was also found to contribute to cyberbullying in 1103 Chinese adolescents (Wang & Ngai, 2022). Studies also explored other common motivations to bully, which include amusement, to have fun or create excitement, to look cool and to increase their power, status, and popularity (Thornberg & Knutsen, 2011; Thornberg et al., 2012), and social dominance (Nassem & Harris, 2015). Therefore, children who bully may be motivated by several factors either independently or in combination, but findings suggest that positive emotion is a factor contributing to bullying, for both the bully as well as ‘reinforcers’ who reward others for bullying through smiling, laughter, and other expressions of positive affect.

### Parent Socialization of Emotion in Relation to Child and Adolescent Antisocial Behavior

Children learn to express, understand and manage emotions based on their parents’ responses to their communication of emotion (Johnson et al., 2017). Parents are theorized to socialize their children’s emotions through three main processes: (a) reactions to their child's emotional displays; (b) discussion of emotion; and (c) emotional expressiveness within the family (Eisenberg et al., 1998). Parenting behaviors that are supportive of children’s emotional expression include encouragement to express emotion, and emotion or problem-focused responses to children’s displays of emotion, with these parenting practices related to better understanding of emotions, more effective emotion regulation, and positive child adjustment (Fabes et al., 1990). In contrast, parents who discourage, minimize, or have punitive reactions to their child’s emotional displays risk socializing their child to suppress their feelings until they are released in a highly intense, dysregulated manner (Buck, 1984). Younger children may be more reliant on parent emotion socialization to support them to regulate their emotions (Fabes et al., 2002). A logical extension to this theory is that children whose parents provide encouragement for the expression of positive affect in the context of AB, or via parental modelling of positive affect in relation to antisocial acts, may be more likely to engage in AB themselves. A meta-analysis by Johnson et al. found that parent emotion socialization-related behaviors were more strongly associated with child conduct problems when parents were focused on negative rather than positive emotions. However, studies tended to assess positive emotion in general (e.g., ‘talk with your child about a time that he or she was happy’; Pasalich et al., 2012), or positive emotion expressed in relation to affiliative or prosocial behavior. One exception was a study by Chaplin et al. (2005) who found that fathers paid more attention to boys’ expressions of ‘disharmonious’ happiness (e.g., laughing at others’ mistakes). Boys may be at higher risk for positive emotion-related AB due to cultural views on gender roles and emotion display rules, coupled with individual vulnerability factors for emotion dysregulation that are more prevalent in boys (e.g., temperament risk, language difficulties) (Brody, 2000). However, to the best of our knowledge, these possibilities are yet to be investigated.

### Peer Influence and the Role of Positive Emotion in Child and Adolescent Antisocial Behavior

Antisocial children are more likely to be rejected by prosocial peers (Davis & Allen, 2023) and to self-select into deviant peer groups (Dishion et al., 2006). Peers have a strong influence on AB, and adolescents are significantly more likely to engage in delinquent acts with antisocial peers (Fergusson et al., 2002). In relation to positive emotion and AB, research on peer influences has chiefly focused on (i) the role of humour in antisocial peer groups, (ii) positive affect experienced when planning, participating in, and/or discussing antisocial acts with peers, and (iii) positive affect as a means of developing and strengthening bonds with peers while participating in shared antisocial acts. A major theory driving research on positive affect, AB and peer process is Dishion’s deviancy training model (Dishion et al., 1996). This model draws on social learning (operant) principles including the modelling and imitation of aggressive behavior (Bandura & Walters, 1997) and the role of reciprocal, interactive coercive exchanges between the child and significant others (e.g., parents, siblings) in reinforcing coercive behaviors (e.g., whining, hitting) and extinguishing warm, positive interactions (Patterson et al., 1975). These reciprocal patterns of coercive interaction may escalate over time and generalize to settings outside of the family (e.g., peers, school). Similarly, in Dishion et al.’s model, peers ‘train’ each other to behave in an antisocial manner through positive responses (e.g., smiles, laughter) in response to actual AB or through the discussion of antisocial acts, which normalizes and encourages these behaviors. Thus children learn to behave antisocially through the observation, imitation and positive reinforcement of deviant attitudes and behaviors, resulting in the initiation and persistence of AB. This theory has strong empirical support and led to greater awareness of the potential harmful effects of peer group interventions for delinquency (Dishion et al., 2006). Peer influence on AB may be heightened during adolescence, a time when issues such as social status, popularity, and peer pressure come to the fore (Steinberg & Monahan, 2007). Adolescence is also characterized by risk-taking due to an increased reward orientation coupled with a lag in cognitive control (Chein et al., 2011). These risk factors may play a stronger role for boys (Centifanti & Modecki, 2013), with the QVT model highlighting the ‘quest for excitement’ as a motivator for the AB of adolescent males, particularly in the peer context and/or when under the influence of alcohol or other substances (McMurran et al., 2010).

### The Current Study

To the best of our knowledge, the only past review exploring the relationship between positive emotion and AB focused solely on adults (Chester, 2017). Given differences in the presentation, prevalence, and severity of AB over the life course (Moffitt, 2018), and developmental changes in emotion understanding and regulation (Eisenberg, 2000), it is important to explore the relationship between positive emotion and AB in children and adolescents. The positive emotion—AB link has been studied in children by different disciplines, including psychology, education, and sociology, with different theories, study designs and methods favoured by these respective fields. Consequently, a systematic review is needed to summarise our current understanding of this phenomenon, and to integrate theoretical perspectives, research findings and guide future research. The aim of this review is to address the following questions: (1) What is the relationship between positive emotion and AB in children and adolescents? (2) Does this relationship differ across different samples, study designs and methods? (3) What individual child or contextual factors interact with positive emotion to influence AB?

## Methods

### Selection of Studies

A protocol for this systematic review was registered on PROSPERO (Moore & Allen, 2022, Prospero registration number: CRD42022298993) and the Preferred Reporting Items for Systematic Reviews and Meta-Analysis (PRISMA; Page et al., 2021) standards were followed. All studies investigating positive affect in relation to AB in children and adolescents that met the inclusion criteria were included (see Table 1 for eligibility criteria). AB was broadly defined, encompassing ODD/CD, conduct problems, delinquency, and associated behaviors: bullying, aggression, vandalism, theft, and illegal acts. Studies on substance use, alcohol use, and gambling were not included, as these are behaviors considered central to substance-related and addictive disorders rather than disruptive behavior disorders. The outcome of interest in the review is positive emotion, which encompasses pleasure, excitement, pride, happiness, joy, amusement, and delight.

**Table 1:** Summary of the eligibility criteria

	Inclusion	Exclusion
Population/participants: Children and adolescents Profile of antisocial behavior	Participants must be aged ≤ 18 years Participants must have a profile of antisocial behavior (as indicated by self and proxy reports)	Participants aged ≥ 19 years Participants without a profile of antisocial behavior Participants with autism or a learning disability
Intervention/exposure: Antisocial behaviour i.e., oppositional defiant disorder (ODD), conduct disorder (CD), conduct problems, delinquency, aggression, vandalism, theft, crime, bullying and illegal acts	Self and proxy reports of antisocial behavior, including standardised measures. All reports of antisocial behavior will be included	Substance use, alcohol use, gambling and playing violent games
Outcome: Positive emotion i.e., pleasure, excitement, pride, happiness, joy, amusement, and delight	Self-report or proxy report of positive emotion associated with antisocial behavior. All reports of positive emotion will be included as there are limited standardised measures of positive emotion	None
Types of study	Quantitative, qualitative or mixed-methods design Written in English Published peer-reviewed articles and unpublished articles	Written in a language other than English

### Search Strategy

A systematic review of the literature was conducted by searching electronic databases for published papers in APA PsycNet, PubMed, Embase, International Bibliography of the Social Sciences (IBSS) and Scopus on 14th January 2023. To identify unpublished research such as theses, dissertations, and pre-prints, the electronic databases PsycEXTRA and EthOS were searched on 14th January 2023. This search was re-run on the 7th April 2024, and revealed an additional 3 articles that met inclusion criteria. Titles, abstracts, and keywords were searched using a combination of free text and index terms relating to antisocial behavior and positive emotion (Appendix A). The ‘AND’ and ‘OR’ Boolean operators were used to compile the search on each database, combining all terms and characteristics for antisocial behavior and positive emotion. Searches were limited to human studies with participants aged 18 years old or younger. There were no limitations on publication date. The reference lists of included articles were inspected manually for any additional relevant studies.

### Screening and Data Extraction

Screening was conducted using Covidence Systematic Review Software (2023). After removing duplicates, all titles and abstracts were reviewed by the first author (J.M.), and 20% were randomly selected to be independently reviewed by the second author (L.T). All full text articles were reviewed independently by the first and second authors. Disagreements were resolved through discussion. If agreement was not reached, the article was discussed with the last author (J.A.), with the majority vote accepted. A data extraction tool designed by J.M. was embedded as a form and piloted within Covidence, with all data manually entered by J.M. This tool was used to collect information on (1) article identification, (2) aims and hypotheses, (3) sample and methodological details, (4) measures and informants of positive emotion, (5) measures and informants of AB, (6) study procedures, (7) findings, and (8) study limitations.

### Quality Assessment

All studies were assessed using the Mixed Methods Assessment Tool (MMAT; Hong et al., 2018). This tool is used to appraise the quality of five types of research: qualitative, randomised-controlled trials, non-randomised studies, quantitative descriptive studies, and mixed methods studies. For each study, there are two screening questions. Responding ‘no’ or ‘can’t tell’ to these questions may indicate that the paper is not an empirical study and cannot be appraised by the MMAT. For each study, the reviewers must select the most appropriate research category and provide five ratings for the chosen category, with the responses being ‘yes’, ‘no’ or ‘can’t tell’. Studies were independently assessed by the first and second authors; any disagreements were resolved through discussion.

### Data Synthesis

The literature search produced research using a wide variety of methodological approaches to explore the relationship between positive emotion and AB in children and adolescents. Given how disparate the studies were in terms of sample, design and methods, a narrative analysis was adopted to synthesise the relevant information.

## Results

### Study Characteristics

**Fig. 1:**
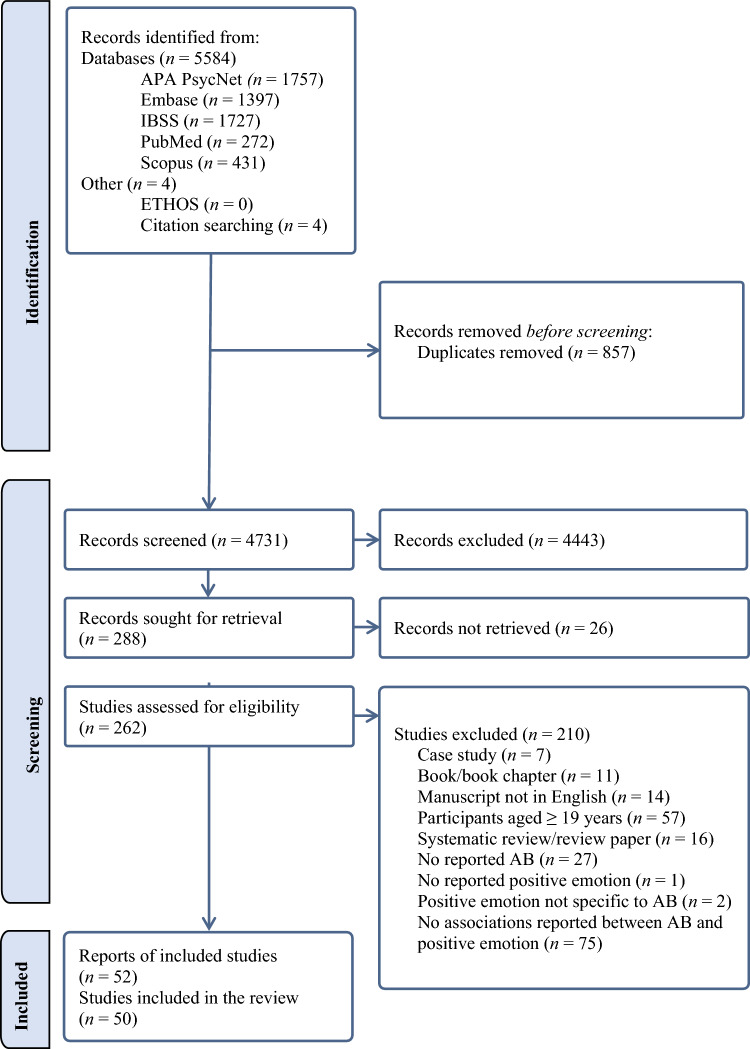
PRISMA diagram

Figure 1 shows the review process using a PRISMA diagram. Fifty studies met the inclusion criteria (52 manuscripts, with 4 manuscripts reporting on overlapping samples). Table 2 presents study details and quality assessment ratings. Sample sizes ranged from *n* = 9 to *n* = 7675, although one paper did not report the sample size (Agnew, 1990). Participant ages ranged from 3 to 18 years old, with most children of school age (5–16 years old). Belson (1976) did not report participant age, but referred to them as ‘juveniles’, indicating that they were under the age of 18. Weenink (2014) reported that participants were aged up to 18 years but did not report the age range. Of the 50 studies included, 32 used community samples, 8 used forensic samples, one used a clinical sample, 5 used community and clinical samples, three used community and forensic samples, and one used a clinical and forensic sample. Thirty-eight studies had a mixed gender sample, and of these studies, three did not report the proportions of girls and boys (Agnew, 1990; Harden et al., 2012; Weenink, 2014). Eight studies had 100% male participants, two had 100% female participants and two did not report gender (Blair, 1997; Warm, 1997).

**Table 2:** Study characteristics, outcomes and quality assessment

Qualitative studies (*n* = 9)										
Authors and year	Sample type	Country	*N*	% female	Sample age range in years (M)	Reports/evidence of AB (informant)	Reports/evidence of positive emotion (informant)	Relationship	MMAT ratings	
Anderson and Linden (2014)	Forensic	Canada	43	5%	(M = 16)	Recruited from juvenile prison	Semi-structured interview (self-report)	Yes (qualitative description)	1.1 Yes 1.2 Can’t tell 1.3 Can’t tell 1.4 Yes 1.5 Can’t tell	
Ash et al. (1996)	Forensic	USA	63	33%	13–18 (M = 15.7)	Recruited from juvenile prison	Semi-structured interview (self-report)	Yes (qualitative description)	1.1 Yes 1.2 Yes 1.3 Can’t tell 1.4 Yes 1.5 Yes	
Belson (1976)	Community	UK	1425	0%	‘juveniles’ but age not explicitly reported	Semi-structured interview (self-report)	Semi-structured interview (self-report)	Yes (qualitative description)	1.1 Yes 1.2 Can’t tell 1.3 Can’t tell 1.4 No 1.5 Can’t tell	
Cao et al. (2023)	Community	China	40	15%	4 to 6 (M = 5.3)	Teacher questionnaire report	Semi-structured interview (teacher report)	Yes (qualitative description)	1.1 Yes 1.2 Yes 1.3 Yes 1.4 Yes 1.5 Yes	
Gabaldón (2021)	Forensic	Venezuela	18	11%	14–17	Recruited from juvenile prison	Semi-structured interview (self-report)	Yes (qualitative description)	1.1 Yes 1.2 Yes 1.3 Yes 1.4 Can’t tell 1.5 Yes	
Hochhaus and Sousa (1987)	Community; Forensic	USA	9	0%	13–16	Gang involvement (self-report or reported by criminal justice system)	Semi-structured interview (self-report)	Yes (qualitative description)	1.1 Yes 1.2 Yes 1.3 Can’t tell 1.4 No 1.5 Can’t tell	
Ojo (2008)	Forensic	USA	20	0%	14–17	Involvement with the criminal justice system and gang involvement (self-report or reported by criminal justice system)	Semi-structured interview (self-report)	Yes (qualitative description)	1.1 Yes 1.2 Yes 1.3 Yes 1.4 Yes 1.5 Yes	
Owens et al. (2000), Shute et al. (2002)*	Community	Australia	54	100%	15–16	Semi-structured focus groups (self-report)	Semi-structured focus groups (self-report)	Yes (qualitative description)	1.1 Yes 1.2 Yes 1.3 Yes 1.4 Yes 1.5 Yes	
Owens et al. (2005, 2007)*	Community	Australia	72	56%	14–15	Semi-structured focus groups (self-report)	Semi-structured focus groups (self-report)	Yes (qualitative description)	1.1 Yes 1.2 Yes 1.3 Yes 1.4 Yes 1.5 Yes	

Quantitative non-randomized studies (*n* = 17)										
Authors and year	Sample type	Country	*N*	% female	Sample age range in years (M)	Reports/evidence of AB (informant)	Reports/evidence of positive emotion (informant)	Comparison groups	Relationship	MMAT ratings
Arsenio et al. (2004)	Community; Clinical	USA	100	31%	Disruptive (M = 16.02) Comparison (M = 15.77)	Existing diagnosis of ODD or CD, or recruited from a specialist school for children with behavioral difficulties (psychologist) RPQ (teacher-report) CBCL-TRF (teacher-report)	Aggression emotion expectancies (self-report) Nonaggression emotion expectancies (self-report) Emotion chip method (self-report)	Disruptive children vs non-disruptive children	Positive, significant	3.1 No 3.2 Yes 3.3 No 3.4 No 3.5 Yes
Benenson et al. (2008)	Community	UK	Sample 1: 89 Sample 2: 335	Sample 1: 0% Sample 2: 38%	4–9	Both samples: Interview (self-report), and responses coded (researcher-coded)	Sample 2: Rating scales (self-report)	Boys (from both samples) vs girls in sample 2	Positive, significant	3.1 Yes 3.2 Yes 3.3 Yes 3.4 Can’t tell 3.5 Yes
Blair (1997)	Clinical	UK	32	Not reported	Children with high psychopathic tendencies (M = 13.20) Children with low psychopathic tendencies (M = 12.79)	Recruited from a school for children with Emotional and Behavioral Difficulties PSD (teacher-report)	Interview in relation to vignettes (self-report)	Children with high psychopathic tendencies vs children with low psychopathic tendencies	Null	3.1 No 3.2 Yes 3.3 Yes 3.4 No 3.5 Yes
Cimbora and McIntosh (2003)	Community; Clinical	USA	63	0%	13–18	Existing diagnosis of CD (clinician), verified by researchers	AMI (self-report) Interview (self-report), and responses coded (researcher-coded)	Diagnosis of CD vs no CD	Positive, significant	3.1 No 3.2 Yes 3.3 Yes 3.4 Yes 3.5 Yes
de Castro et al. (2005)	Community; Clinical	Nether-lands	84	0%	7–13 (M = 10.10)	Aggressive ppts were recruited from behavior disorder clinics and specialist schools for children with behavior problems TRF- Dutch version (teacher-report) RPQ (teacher-report)	Interview, rating scales, vignettes (self-report). Responses were coded (researchers)	Aggressive vs non-aggressive boys	Positive, significant	3.1 No 3.2 Yes 3.3 Yes 3.4 Yes 3.5 Yes
Dishion et al. (1996)	Community	USA	206	0%	13–14	Peer interaction task (researchers) Police contact (juvenile court records) National Youth Survey measure of self-reported delinquency (self-report)	Peer interaction task (researchers)	Delinquent dyads vs non-delinquent dyads vs mixed dyads	Positive, significant	3.1 Yes 3.2 Yes 3.3 Yes 3.4 Yes 3.5 Yes
Gasser et al. (2012)	Community	Switzer-land	139	41%	7–9	Peer-nomination scale (peer-report) Children were classified as overtly aggressive or not (researcher)	Interview (self-report). Responses were coded (researchers)	Aggressive vs non-aggressive children	Positive, significant	3.1 No 3.2 Yes 3.3 Yes 3.4 No 3.5 Can’t tell
Gutzwiller‐Helfenfinger and Perren (2021)	Community	Switzer-land	331	51%	(M = 14.9)	Offline bullying measure (self-report) Cyberbullying measure (self-report)	Assessment of moral functioning and emotion expectation using vignettes (self-report)	Open situation: Ashamed moralists vs happy moralists vs indifferent moralists vs happy transgressors Accomplished situation: moralists vs happy opportunists	Positive, significant	3.1 No 3.2 Yes 3.3 Yes 3.4 No 3.5 Yes
Lyon (2001) (Study 3)	Community	USA	79	42%	5–6 (kindergarten) 9–10 (4th grade)	CABI (teacher-report)	Moral feelings task (self-report) and responses coded (researcher)	Aggression: high vs low vs average Depressive: high vs low vs average	Positive, significant	3.1 No 3.2 Yes 3.3 Yes 3.4 No 3.5 Yes
Menesini et al. (2003)	Community	Spain; Italy	179	50%	9–13	Participant roles questionnaire (self-report) and pro-bullying scale (assigned to groups by researchers)	Interview based on the Scan Bullying Test (self-report). Responses were coded (researchers)	Bullies vs victims vs outsiders	Positive, significant	3.1 Yes 3.2 Yes 3.3 No 3.4 No 3.5 Yes
Panayiotou et al. (2015)	Community	Cyprus	91	41%	(M = 11.90)	BVQ-R (self-report) and assigned to groups (researchers) ICU (self-report)	Subjective emotion ratings in response to affective imagery stimuli (self-report)	Bullies vs bully-victims vs victims vs controls	Positive, non-significant	3.1 No 3.2 Yes 3.3 Can’t tell 3.4 No 3.5 Yes
Roos et al. (2011)	Community	Finland	378	56%	(M = 11.30)	Peer reports of aggression (peer-reported)	Anticipated emotional response ratings to vignettes (self-report)	Girls vs boys; high vs low aggression	Positive, non-significant	3.1 Can’t tell 3.2 Yes 3.3 No 3.4 No 3.5 Yes
Schalkwijk et al. (2016)	Community; Forensic	Nether-lands	334	41%	13–18 Delinquents (M = 15.53) Controls (M = 14.52)	Delinquent participants recruited from juvenile prison	TOSCA-A (self-report) MOM (self-report) IRI (self-report) CoSS (self-report)	Delinquent vs non-delinquent	Positive, significant	3.1 No 3.2 Yes 3.3 Yes 3.4 Yes 3.5 No
Spidel et al. (2011)	Forensic	Canada	60	25%	12–18 (M = 15.02)	Participants were recruited from juvenile prison PCL:YV (completed from file information by researchers)	Lie identification and deceptive motivations (identified by researchers from file and interview reviews)	Psychopathy: high vs medium vs low	Positive, significant	3.1 No 3.2 Yes 3.3 Yes 3.4 No 3.5 Yes
van Dijk et al. (2017)	Community	Nether-lands	283	41%	4–9 (M = 6.70)	Peer-nomination interview (peer report) IRPA (teacher-report)	Interview (self-report). Responses were coded (researchers) IRPA (teacher-report)	Bully vs bully-victim vs non-involved	Null	3.1 No 3.2 Yes 3.3 No 3.4 Yes 3.5 Yes
Vylegzhanina et al. (2017)	Community	Russia	228	52%	8–9	Modified MVB (parent-report)	Modified MVB (parent-report)	Propensity for vandalism: high vs medium vs low	Positive, significant	3.1 No 3.2 Yes 3.3 Can’t tell 3.4 No 3.5 Yes
Warm (1997)	Community	USA	250	Not reported	6–17 1st grade: (M = 7) 3rd grade: (M = 9) 6th grade: (M = 12) 8th grade: (M = 14) 11th grade: (M = 17)	Structured interview (self-report). Responses were coded (researchers)	Structured interview (self-report). Responses were coded (researchers)	Age/grade: 1st grade vs 3rd grade vs 6th grade vs 8th grade vs 11th grade	Positive, significant	3.1 Yes 3.2 Yes 3.3 No 3.4 No 3.5 Yes

Quantitative descriptive studies (*n* = 21)										
Authors and year	Sample type	Country	*N*	% female	Sample age range in years (M)	Reports/evidence of AB (informant)	Reports/evidence of positive emotion (informant)	Relationship	MMAT ratings	
Agnew (1990)	Community	USA	Not reported	Not reported	11–18	Semi-structured interview (self-report). Responses were coded (researchers)	Semi-structured interview (self-report). Responses were coded (researchers)	Yes (descriptive statistics were used, significance not reported)	4.1 Can’t tell 4.2 Yes 4.3 Can’t tell 4.4 Can’t tell 4.5 Can’t tell	
Aluja-Fabregat and Torrubia-Beltri (1998)	Community	Spain	470	50%	(M = 13.64)	EPQ (self-report) PTSBS (teacher-report)	SSS (self-report) PTSBS (teacher-report) Ratings given to violent cartoons (self-report)	Positive, significant	4.1 Yes 4.2 Can’t tell 4.3 Yes 4.4 Yes 4.5 Yes	
Arsenio et al. (2009)	Community	USA	100	31%	(M = 15.90)	Diagnosis of CD/ODD or no diagnosis (psychologists) CBCL-TRF (teacher-report) MRPA (teacher-report)	Anticipated response and outcome expectancies based on vignettes (self-report) Adolescents moral reasoning task (self-report)	Positive, significant	4.1 Can’t tell 4.2 No 4.3 Yes 4.4 Yes 4.5 Yes	
Arsenio et al. (2000)	Community	USA	51	53%	3–5 (M = 4)	Observation (researchers) Aggression scale (teacher-report) Peer ratings of their aggression (peer-report)	Observation (researchers)	Positive, significant	4.1 Yes 4.2 No 4.3 Yes 4.4 Yes 4.5 Yes	
Arsenio and Lover (1997)	Community	USA	37	49%	4–5	Observation (researchers)	Observation (researchers)	Positive, significant	4.1 Can’t tell 4.2 No 4.3 Yes 4.4 Yes 4.5 Yes	
Arsenio and Ramos-Marcuse (2014)	Community; Clinical	USA	63	62%	3–6 (M = 4)	CBCL (parent-report) Caregiver-Teacher Report Form (therapist-report)	MSSB (self-report). Responses were coded (researchers)	Null	4.1 Yes 4.2 Yes 4.3 Yes 4.4 Can’t tell 4.5 Yes	
Bjørnebekk and Howard (2012)	Clinical; Forensic	Norway	101	37%	12–18 (M = 15)	Met criteria for any behavior listed in the DSM-IV for ODD, CD or disruptive behaviors such as aggression or delinquency (welfare authorities) TRF (teacher-report) SRD (self-report)	AAS (self-report)	Positive, significant	4.1 Yes 4.2 Yes 4.3 Yes 4.4 Yes 4.5 Yes	
Byck et al. (2015)	Community	USA	592	51%	13–18 (M = 15.9)	Mobile Youth Survey (self-report)	SSS (self-report)	Positive, significant	4.1 Yes 4.2 Yes 4.3 Yes 4.4 No 4.5 Yes	
Callender et al. (2010)	Community; Clinical	USA	215	47%	Time 1 (M = 5.5) Time 2 (M = 10)	CBQ (parent-report) CBCL (parent-report) TRF (teacher-report) Experimental task. Children’s behaviors were coded to create a ‘cheating severity score’ (researchers)	Experimental task. Children’s behaviors were coded for inappropriate positive affect (researchers)	Yes (descriptive statistics were used, significance not reported)	4.1 Yes 4.2 Yes 4.3 Yes 4.4 Yes 4.5 Yes	
Ching et al. (2014)	Forensic	Australia	143	22%	(M = 16)	PCL:YV (completed from case file information by researchers)	Function of index offences from case files were coded (researchers)	Positive, significant	4.1 Yes 4.2 Yes 4.3 Yes 4.4 No 4.5 Yes	
Erreygers et al. (2017)	Community	Belgium	1720	54%	(M = 13.61)	Measure of engagement in prosocial and antisocial behavior online (self-report)	Measure of emotions in the past month (self-report)	Null	4.1 Yes 4.2 No 4.3 Yes 4.4 No 4.5 Yes	
Feilhauer et al. (2013)	Community	Belgium	46	0%	8–12	YSR (self-report) ICU (self-report)	AMI (self-report) Two additional structured questions about emotion (self-report)	Positive, significant	4.1 Yes 4.2 No 4.3 Yes 4.4 Yes 4.5 Yes	
Harden et al. (2012)	Community	USA	7675	Not reported	Time 1: 10–11 Time 2: 17	SRD (self-report)	3-item measure of sensation seeking (self-report)	Positive, significant	4.1 Yes 4.2 Yes 4.3 Yes 4.4 No 4.5 Yes	
Hasegawa (2023)	Community	Japan	371	48%	9–13	Bullying Behavior Scale (self-report)	Judgment Related to Peer Exclusion using vignettes (self-report) One item on emotion attribution (self-report)	Positive, significant for Judgement Null for emotion attribution	4.1 Yes 4.2 Can’t tell 4.3 Yes 4.4 Can’t tell 4.5 Yes	
Hawes et al. (2019)	Community	Australia	90	0%	10–13 (M = 11.29)	APSD (parent-report) SDQ (parent-report)	Experimental task (young person), and ratings of their emotional states (self-report)	Positive, significant	4.1 Yes 4.2 No 4.3 Yes 4.4 No 4.5 Yes	
Kunimatsu et al. (2012)	Forensic	USA	58 (60)	100%	12–18 (M = 14.98)	Participants were recruited from juvenile prison PCS (self-report) ICU (self-report) Adolescent Stories Interview (self-report) SRD (self-report)	Adolescent Stories Interview (self-report)	Positive, significant	4.1 Yes 4.2 No 4.3 Yes 4.4 Yes 4.5 Yes	
Marrington et al. (2023)	Community	Australia	157	58%	13–18 (M = 15.58)	Global Assessment of Internet Trolling-Revised (self-report)	Social Rewards Questionnaire (self-report)	Positive, significant	4.1 Yes 4.2 No 4.3 Yes 4.4 Can’t tell 4.5 Yes	
Mishna et al. (2010)	Community	Canada	2186	55%	11–17	Questionnaire about experience of cyberbullying (self-report)	Questionnaire about experience of cyberbullying (self-report)	Yes (qualitative description)	4.1 Yes 4.2 Yes 4.3 Yes 4.4 No 4.5 Yes	
Ortiz Barón et al. (2018)	Community	Spain	351	56%	10–14 (M = 12.25)	Antisocial Behavior scale of the BPQ (teacher-report)	AMP (self-report) TOSCA-C (self-report)	Null	4.1 Yes 4.2 Yes 4.3 Yes 4.4 Can’t tell 4.5 Yes	
Wong et al. (2023)	Community	Canada	150	50%	4 -5 (M = 4.53)	Three items from the aggression/ hostility scale of the Berkeley Puppet Interview (self-report) Six items from the Child Behavior Checklist (parent-report)	Seven vignettes from the Social-Emotional Responding Task (self-report, and responses coded by the researchers)	Positive, significant	4.1 Can’t tell 4.2 Can’t tell 4.3 Yes 4.4 Yes 4.5 Yes	
Wong and McBride (2018)	Community	Hong Kong	750	52%	11–15	Measure of cyberbullying perpetration and victimization (self-report)	Fun-Seeking subscale of the BAS (self-report) NBCA (self-report)	Positive, significant	4.1 Yes 4.2 Yes 4.3 Yes 4.4 Can’t tell 4.5 Yes	
*Mixed methods studies (N* = *3)*										
Allen et al. (2018)	Community	UK	47	51%	11 to 14 (M = 12.50)	Teachers and child self-report	Teacher semi-structured interview on the top 25% on student-report CU score (*n* = 24) and below the median ICU score (*n* = 23)	Yes (qualitative description)	5.1 No 5.2 Yes 5.3 Yes 5.4 Can’t tell 5.5 Yes	
Walker-Barnes and Mason (2001)	Community; Forensic	USA	31	100%	12 to 17 (M = 14.79)	Participants recruited from a specialist school for girls at high risk for delinquency	Semi-structured interview (self-report)	Yes (qualitative description)	5.1 Yes 5.2 Yes 5.3 Yes 5.4 No 5.5 No	
Weenink (2014)	Forensic	Netherlands	159	Not reported	Up to 18 (but did not report age range)	Perpetration of a violent offence (judicial files). Offences were coded as ‘frenzied’ or not (researchers)	Judicial files containing perpetrator reports (self-report). Emotions were coded (researchers)	Yes (qualitative description)	5.1 No 5.2 Yes 5.3 Yes 5.4 No 5.5 No	

Nine studies used qualitative methods, 17 were quantitative non-randomized studies, 21 used quantitative descriptive methods, and three used mixed methods. Forty-four studies were cross-sectional, and six were longitudinal studies. AB took the form of either overt aggression (including relational and proactive aggression) or reactive aggression, bullying, or other specific forms of AB (e.g., theft, deceit, and vandalism) or hypothetical scenarios of antisocial acts. Informants for AB included children, peers, parents, teachers, and clinicians, with other sources including criminal records, or the sample was defined as antisocial based on the recruitment setting (e.g., juvenile prisons, special education schools, clinics). AB was assessed via questionnaires, interviews, observational methods (e.g., peer interaction, temptation tasks) or experimental tasks. Informants of positive emotion included children, parents or teachers via questionnaires, interviews, and self-ratings during experimental tasks. Independent raters also provided ratings of positive affect based on child responses to hypothetical vignettes or story stems, or judicial or prison file details. Observer ratings of child emotions during activities or experimental tasks were also used (e.g., type and intensity of facial emotion expressions). There was considerable overlap between quantitative and qualitative studies for informants and assessment methods. However, experimental tasks, behavior observation methods and hypothetical scenarios were confined to quantitative studies, while focus groups were exclusive to studies with a qualitative component.

### Overt Aggression and Positive Emotion

Thirteen papers described the relationship between overt aggression and positive emotion, with all studies finding a positive relationship between these two constructs in community, forensic and clinical populations.

#### Overt Aggression and Positive Emotion in Community Samples

One study (Arsenio & Lover, 1997) observed children’s emotions during disputes with same-age peers in children aged 4–5 years old. Children were more likely to show negative emotions during physical aggression, but some children also displayed happiness at medium or high intensity. Children’s baseline happiness and aggression-related happiness were unrelated. However, aggression-related happiness predicted increased aggression and initiation of conflict, even when accounting for baseline levels of anger and happiness. Aggression-related happiness was related to lower peer acceptance, and the recipients of aggression displayed more distress when the aggressors were happy (Arsenio et al., 2000; Arsenio & Lover, 1997). One study also using observation methods (Benenson et al., 2008) found that 50% of boys and less than 10% of girls experienced pleasure from enacting physical aggression with their toys. Similarly, more boys enjoyed aggressive play with their peers compared to girls. Positive emotion in relation to overt aggression was also found in older children aged 11 to 18 years from a community sample (Agnew, 1990). The most common self-reported reasons for engaging in property crime, vandalism, illegal entry, and trespassing included self-gratification, pleasure seeking, curiosity and thrills.

#### Overt Aggression and Positive Emotion in Clinical and Forensic Samples

Evidence from studies using clinical and forensic samples suggests that some youth expect planned antisocial acts to lead to positive emotion. One study found that antisocial adolescents expected to feel happier following acts of proactive aggression (Arsenio et al., 2004). When reactive aggression was controlled for, greater proactive aggression was still related to greater self-reported happiness. In contrast, when ratings of proactive aggression were controlled for, reactive aggression was not significantly related to positive emotion. One study examined four motivationally distinct types of aggression based on the QVT (explosive/reactive, thrill-seeking, coercive, and vengeful/ruminative) in a combined clinical and forensic sample of adolescents (Bjørnebekk & Howard, 2012). Thrill-seeking was a significant predictor of AB, particularly in relation to vandalism, carrying hidden weapons and violent offending even after controlling for the other types of motivation. Another study (Ching et al., 2014) found that appetitively violent acts were significantly more likely to be perpetrated in a group context and by males, with 13.5% of boys anticipating that a violent act would enhance their mood.

The qualitative literature on gang membership aligns with quantitative research findings, indicating that some young people are at least partly motivated to engage in overt aggression as part of a gang because they anticipate positive affect as a consequence. Young people in forensic samples indicated that they engage in overt aggression because ‘it’s a fun thing to do’ (Ojo, 2008), with one study finding that 68% of their sample believed that gangs provide adventure (Walker-Barnes & Mason, 2001), and 40% of young people reported feeling energised, excited, or powerful when carrying a gun (Ash et al., 1996). In one qualitative study, interviews with young people incarcerated for homicide revealed that the two main emotions associated with their crime were negative (Gabaldón, 2021). Fear was associated with the aim to defend themselves, while rage was accompanied by positive emotions including a sense of power, excitement, physical arousal, and adrenaline. Weenink reported that solidarity excitement, defined as an intense shared feeling of excitement that provides a strong sense of solidarity within the group, can emerge prior to a planned violent attack. Several adolescents reported ‘we went for the thrill of it’. One stated, ‘I was enjoying it at the time… had a laugh’. Two participants also experienced positive emotions following the violent act, reporting feeling ‘proud’ of it, and telling their friends about this ‘cool story’. Solidarity excitement appeared more often prior to frenzied attacks, particularly if there was already a strong consensus within the group to commit violence. Another qualitative study of adolescents also found that excitement was identified as one of the most frequent motivations for joining a gang (Hochhaus & Sousa, 1987).

Findings from qualitative studies also indicated that the desire to attain social status, social dominance and respect from peers were also motivations for joining a gang and engaging in gang-related activities, including aggression. One youth reported feeling like a ‘superhero, the owner of the world and the best of all of them’. Several participants reported enjoying the sense of power and respect they receive from being in a gang: ‘I feel good to be part of the gang’, ‘I wanted power and fame… and respect’ and ‘gang members look cool, and I like the attention they get’ (Hochhaus & Sousa, 1987; Ojo, 2008; Walker-Barnes & Mason, 2001).

### Relational Aggression and Positive Emotion in Community Samples

Four studies found that relational aggression (i.e., harming someone’s relationships or social position) was associated with positive emotion in community samples (Owens et al., 2000, 2005, 2007; Shute et al., 2002). One qualitative study of adolescent girls focused on non-verbal expressions of social aggression, and two subcategories of relational aggression, which included non-verbal behaviors such as aggressive stares, gestures, sarcasm, and exclusionary behaviors (Shute et al., 2002). One girl described this behavior as ‘fun’, with several others reporting they engaged in these behaviors either to create excitement or to alleviate boredom. In two qualitative studies using the same sample (Owens et al., 2000, 2005) the most common explanation for social aggression was for fun or excitement, particularly in relation to spreading rumours, talking about others unkindly behind their back, and bringing down someone’s confidence. Boys and girls agreed they ‘do it to get a laugh’, with one boy reporting ‘maybe some people just want to have a joke once in a while… it’s a good laugh’. However, others described their motivation for gossiping as simply ‘for something to do’ to overcome boredom. Boys reported there was ‘not much’ verbal aggression from them towards girls, however when there was, it was because they were ‘only joking’. Other motivations for relational aggression (except revenge) did not suggest a central role of positive emotion, such as wanting to impress others, to gain acceptance from peers, and peer pressure.

### Bullying and Positive Emotion in Community Samples

Six of the 52 studies using community samples described the relationship between bullying and positive emotion. One study found that positive emotions were not significantly related to online AB, but this relationship was mediated by media use (Erreygers et al., 2017), such that the more emotional adolescents were (either positive or negative), the more they turned to media such as instant messaging, watching videos, or playing online games. Thus, if adolescents experienced intense positive emotions, they also performed and received more online AB, but only if they turned to social and audio-visual media. It was suggested when individuals display too much of their affective state online, they are less likely to be liked and to receive social support (Bellur et al., 2008; Forest & Wood, 2012). Erreygers et al. suggested that this may be why sharing emotions online may elicit antisocial responses from others, and possibly in turn, lead adolescents to behave antisocially themselves.

The other five studies found relationships between bullying and positive emotion. One study identified different types of teasing: hurtful teasing (physical aggression), mean teasing (insults or profanity) and symbolic teasing (using words and gestures; Warm, 1997). The most common reason for teasing given by children aged 6–17 years was because ‘it is fun’. Further elaboration revealed that teasers experienced pleasure in the misery of the victim. Fun, along with other reasons such as revenge and drive for power comprised 80% of the motives for teasing. In one mixed methods study (Allen et al., 2018) high school teachers reported that children high in CU traits and disruptive behavior appeared to enjoy others’ distress: ‘It’s her picking what she says to somebody to get a reaction out of them because she finds some kind of joy in doing it.’ Likewise, in a qualitative study by Cao et al. (2023), Chinese teachers reported that preschool children high in CU traits and disruptive behavior were more likely to make fun of others than children with disruptive behavior low in CU traits: ‘If any children wet their pants, we teachers help them to change clothes…. He would come over every time, laughing and shouting something like ‘Hey, look! He pissed his pants again!’. The remaining two studies investigated cyberbullying in community samples. In a Canadian study (Mishna et al., 2010), children reported feeling like they were funny (25%), powerful (9%), popular (6%), or better than others (4%). Similarly, in a Hong Kong-based study (Wong & McBride, 2018), self-reported fun-seeking was significantly related to increased cyberbullying.

### Positive Emotion and Other Forms of Antisocial Behavior

Seven studies found a significant relationship between the anticipation of positive emotion resulting from engaging in different forms of AB in community, forensic and clinical samples.

#### Positive Emotion and Other Forms of Antisocial Behavior in Community Samples

In an experimental study with 5 year olds (Callender et al., 2010), 54% of children who cheated on an experimental task displayed positive affect (i.e., smiling, grinning, or laughing) during or after cheating. Positive affect displayed when cheating was also related to greater impulsivity and lower inhibitory control. This study also explored longitudinal associations between cheating severity at age five, and externalising symptoms at age 10. Children who displayed positive affect when cheating had more severe externalizing problems at home and school between the ages of 6–10 years old. This suggests that experiencing positive affect when cheating may contribute to the persistence of externalizing problems over time.

One study found that young people reported stealing for various reasons, including for fun and excitement, because their peers were already stealing, the belief that police would not catch them, and a permissive attitude towards stealing (Belson, 1976). Another study investigating motivations for vandalism in a community sample found that some acts were driven by children’s desire for new experiences (Vylegzhanina et al., 2017). The possession of new things increased positive affect, which children sought to re-experience by changing the appearance of their belongings items via vandalism. One Australian study explored adolescent online trolling (Marrington et al., 2023), conceptualised as online AB intended to provoke and distress others for one’s own amusement, and is considered different to cyberbullying. Boys were more likely to troll others, and trolling was significantly related to lower cognitive empathy, higher levels of psychopathy, sadism and negative social potency (i.e., the degree to which one experiences reward when engaging in antisocial interpersonal interactions).

#### Positive Emotion and Other Forms of Antisocial Behavior in Clinical and Forensic Samples

Studies examining auto-theft, rule breaking, and deception also found that positive emotion motivated and/or reinforced these behaviors. A study exploring deviancy in male adolescent friendships (Dishion et al., 1996) found that delinquent dyads reacted more positively to rule-breaking topics of conversation, and were less likely to reinforce prosocial discussions. Delinquent dyads and mixed-dyads (one delinquent boy and one non-delinquent boy) laughed significantly more often in response to rule-breaking topics, compared to non-delinquent dyads. Furthermore, boys in friendships that provided positive responses to rule-breaking conversations escalated their delinquent behavior over the following two years, compared to the other groups that did not react positively to deviant talk, suggesting that positive emotions play a role in peer group processes and the persistence of AB.

In one study investigating youth motives for auto-theft (Anderson & Linden, 2014), the most common reasons included joyriding (93%), for transportation (87%) and for the thrill of it (84%). In fact, 54% of young people said they competed in contests to see who could steal the most cars in one night. Despite concerns about getting caught, 17% said they would still try to steal vehicles ‘for the rush’. One participant reported ‘after you drive when you’re not supposed to drive it’s kind of like an addiction because the adrenaline rush is so powerful. It’s kind of like you always want that rush’. Another study investigated the relationship between motivation to deceive others and psychopathic traits in young offenders (Spidel et al., 2011). Motivations for lying included to obtain a reward, to heighten self-preservation, and to dupe others which provided a sense of delight. Individuals higher in psychopathic traits were more likely to lie for these reasons than those lower in psychopathic traits.

### Antisocial Behavior in Hypothetical Scenarios and Positive Emotion

Eighteen papers described positive emotion in hypothetical scenarios of AB, with 14 reporting positive relationships between these two constructs in community, forensic and clinical samples. Four studies explored emotions in hypothetical bullying situations, 8 studies explored the emotional processes involved during antisocial acts, and 6 studies explored both emotional and cognitive processes involved in hypothetical antisocial acts.

#### Antisocial Behavior in Hypothetical Scenarios and Positive Emotion in Community Samples

Overall, evidence from studies employing hypothetical situations in community samples indicates that there is a significant relationship between positive affect and increased AB. One study used vignettes to examine the development of happy victimisation from early to middle childhood (Wong et al., 2023). Participants were tested at four time points between the ages of four and eight years old. The results indicated the happy victimiser phenomenon was present throughout this period, with happy victimising at Time 1 (age 4) being significantly positively correlated with happy victimising only at Time 2 (age 5). Interestingly, happy victimising at Time 4 (age 8) was significantly related to increased aggression based on child, but not parent-report.

One study using an affective imagery paradigm to evoke emotions (anger, joy, fear, and pleasure-relaxation) found that bullies reported higher positive affect during provocative anger scenes than victims of bullying, and bullying was significantly related to greater joy in response to anger imagery (Panayiotou et al., 2015). Aluja-Fabregat and Torrubia-Beltri (1998) showed children clips from violent cartoons and asked them to evaluate these scenes. Boys enjoyed these films significantly more than girls, with boys rating the seven violent scenes from cartoons as being more amusing and thrilling. The more aggressive boys tended to watch more violent films and rated them as more enjoyable. Another study in Spain and Italy presented children with cartoons portraying bullying scenes and asked how they would feel if they were the bully (Menesini et al., 2003). Children in the bullying group reported significantly greater pride and moral disengagement compared to victims and non-involved children. Many children in the bullying group reported anticipated positive affect due to their actions such as ‘I would feel great because I got the attention of the other children’. Similarly, another study found that ‘happy transgressors’ and ‘happy opportunists’ (i.e., children who do not proactively seek to bully others, but will participate in bullying and experience happiness if this opportunity arises), feel happy about their hypothetical transgressions including assisting the bully, offline and online bullying, and moral disengagement (Gutzwiller-Helfenfinger & Perren, 2021). A study in Japan (Hasegawa, 2023) investigated the relationship between self-reported bullying and how children judged peer exclusion situations, and their emotion attributions using hypothetical scenarios. Children were more likely to judge the exclusion of violent characters to be more acceptable than the exclusion than shy characters, with eighth-graders attributing happy feelings to the excluders significantly more frequently than younger children in fourth or sixth grade. Self-reported bullying was significantly predicted by female gender and negative judgement towards a violent character, but not by emotion attributions. However, another study found that there were no significant differences between bullies, bully-victims and non-involved children for happy-victimiser emotions (van Dijk et al., 2017).

One study using a hypothetical scenario (Lyon, 2001) found that aggressive children were more likely to attribute a positive emotion to a child character who pushes another child, or to a child character who steals from another child than non-aggressive children. These children expected the victimiser to feel gratification from pushing the other child, or to feel happy because they succeeded in obtaining candy. Interestingly, there were no differences between groups in terms of the attributions of positive emotion to a victimiser engaging in relational aggression. Roos et al. (2011) presented children with vignettes describing different conflict situations that resulted in child aggression. They found that children’s anticipated experience of positive emotions varied depending on their gender and peer affiliation. Boys reported more pride and less guilt and shame than girls following aggression. Children whose aggressive friends witnessed their own aggressive actions were more likely to anticipate greater pride, and less guilt and shame than children whose prosocial friends observe their aggression.

One study exploring the relationships between emotion and proactive aggression in 9th–12th graders (Arsenio et al., 2009) found a strong relationship between proactive aggression and expectations that they would feel good after aggressive responses to provocation. Youth with positive outcome expectations for aggression and low moral concern felt greater happiness following unprovoked aggression, and found it easier to act aggressively. Similarly, youth who expected to feel happier following proactive aggression felt it would be easy to respond aggressively to provocation, although they were more likely to explain their positive emotions in relation to material and other gains from aggression. However, adolescents who scored highly on moral reasoning were less likely to report positive emotions following provoked or unprovoked aggression and found it harder to enact aggression, indicating that cognitive processes should be considered in conjunction with affective processes.

In a study of 1st and 3rd graders, children were presented with stories of aggressive transgressions and asked about their thoughts and feelings in relation to the stories (Gasser et al., 2012). Younger aggressive children attributed happiness to the unprovoked aggressor and retaliator, while younger non-aggressive children only attributed happiness to the retaliators. Within the older children, aggressive children were less likely than non-aggressive children to refer to moral reasons in justifying positive emotion attributions and more likely to view retaliation as justified. Feilhauer et al. (2013) found that CU traits and externalising symptoms were related to happiness when imagining having committed antisocial acts, with CU traits also related to less guilt and more excitement. CU traits and externalising symptoms accounted for a significant proportion (17%) of feelings of happiness following imagining an antisocial act. In contrast, internalising symptoms on their own, or combined with CU traits were unrelated to happiness. In an experimental study, Hawes et al. (2019) asked children to play the Iterated Prisoner’s Dilemma. Boys rated by their parents as higher in CU traits were more likely to experience pride following their own defection (i.e., betray and not co-operate with their co-player) in the game.

#### Antisocial Behavior in Hypothetical Scenarios and Positive Emotion in Clinical and Forensic Samples

One study used vignettes to compare children with child-onset (CO) and adolescent-onset (AO) CD and a non-CD group (Cimbora & McIntosh, 2003). Participants were presented with vignettes and asked how happy, excited, afraid, guilty, angry, and ‘other’ they believed the transgressor would feel. They found youth with CO-CD attributed significantly more excitement and happiness to the transgressor than the comparison group, but youth with AO-CD did not differ significantly from the comparison group. CO-CD youth reported the lowest guilt, followed by AO-CD, then the comparison group suggesting the CO-CD group experienced more positive emotional responses. Similarly, in another study using vignettes (de Castro et al., 2005), aggressive boys with behavior problems attributed significantly more hostile intent and happiness to the provocateur and generated more aggressive responses compared to boys from a community sample. Some aggressive boys reported getting fun, glee or ‘kicks’ out of aggression such as coercing others, taking revenge or ‘punishing’ others. In a study featuring a forensic sample of adolescent girls, participants were presented with vignettes and asked questions about the scenarios (Kunimatsu et al., 2012). Happy-victimisation was significantly positively related to CU traits, total delinquency, violent and non-violent delinquency. Happy-victimisation significantly predicted violent delinquency and total delinquency when there were high levels of callousness, but this relationship was not significant at low levels of callousness.

In contrast to the findings of other studies using hypothetical scenarios, one study including both a community and clinical sample (Arsenio & Ramos-Marcuse, 2014) found that while children expected to feel happy following 80% of their moral transgressions, their moral emotion attributions were unrelated to their aggressive tendencies. Children with higher levels of happy victimisation were significantly more likely to provide material reasons (e.g., he got the fancy bike) than positive affect to justify the transgression, but this was not associated with their level of aggression. Interestingly, another study (Blair, 1997) reported that while children with psychopathic tendencies were more likely to judge moral transgressions as acceptable if there were no rules prohibiting the transgression compared to controls, these two groups did not differ significantly in their emotion attributions to the hypothetical stories on happiness, embarrassment, or fear in response to moral transgressions.

### Positive Emotions, Antisocial Behavior and Personality Traits

Six of the 52 studies explored the relationship between AB, positive emotions and personality traits. One study found that offenders reported significantly greater pride following AB compared to a community sample of youth (Schalkwijk et al., 2016). In contrast, another study using a community sample found that AB was unrelated to pride (Ortiz Barón et al., 2018). Three studies explored the relationship between sensation-seeking and AB, with all reporting significant positive relationships. One study asked children to watch brief clips from violent cartoon films and to evaluate these scenes (Aluja-Fabregat & Torrubia-Beltri, 1998). Children who scored higher on sensation-seeking and reward sensitivity were also rated as more aggressive by their teachers. Other traits including psychoticism, disinhibition, and sensation-seeking were related to funny or exciting ratings in boys and girls. Psychoticism was more closely related to the viewing and enjoyment of action-oriented films, whereas sensation-seeking was more related to amusement and excitement in response to violent cartoons. Two longitudinal studies exploring sensation-seeking and AB (Byck et al., 2015; Harden et al., 2012) found that sensation-seeking was significantly positively related to delinquency from childhood to adolescence. While sensation-seeking significantly predicted future delinquency, delinquency did not significantly predict future sensation-seeking suggesting that sensation-seeking drives later delinquent behavior, but not vice versa. Byck et al. (2015) found that girls who showed increased conduct problems also had higher scores on the pleasure-seeking and danger/novelty, whereas this was not observed in boys. Thrill-seeking was not associated with any baseline or growth in conduct problems for girls or boys. One mixed methods study (Allen et al., 2018) indicated that teachers perceived students high in CU traits as displaying positive emotions when engaging in dangerous activities relative to those low in CU traits: ‘He likes new and dangerous things.’

## Discussion

This systematic review identified 52 studies (four of which had overlapping samples) that investigated the relationship between positive emotion and AB in children and adolescents. The vast majority reported that positive emotion was related to AB, and this finding was consistent across community, forensic and clinical samples, and held regardless of whether AB was assessed using real-life antisocial acts or hypothetical scenarios. The consistency of this finding was remarkable given the great in diversity in sample, design and methods in the identified studies. Adult theory highlights the role of positive emotion in relation to the anticipation of, during, and after the antisocial act, predicting that positive emotion may form a positive feedback loop (Chester & DeWall, 2017; Gollwitzer & Bushman, 2012). Our findings provide support for this theory in relation to a variety of forms of AB, with children anticipating positive emotion when engaging in overt aggression (Arsenio et al., 2004; Ching et al., 2014), relational aggression (Shute et al., 2002), bullying (Warm, 1997), theft (Anderson & Linden, 2014; Belson, 1976), lying (Spidel et al., 2011), cheating (Callender et al., 2010) and vandalism (Vylegzhanina et al., 2017). Similarly, studies indicated that children may experience a broad range of AB-related positive emotions including happiness (Arsenio & Lover, 1997), excitement (Gabaldón, 2021; Weenink, 2014), pleasure (Benenson et al., 2008), and a sense of ‘fun’ (Ojo, 2008; Shute et al., 2002; Warm, 1997). Youth also experienced positive emotions following AB, with children reporting feeling ‘proud’ (Hawes et al., 2019; Weenink, 2014), or an increased sense of power, fame, and respect following AB (Hochhaus & Sousa, 1987; Ojo, 2008; Walker-Barnes & Mason, 2001). Consistent with theory (Chester, 2017), positive emotions linked to AB tended to reflect moderate to high rather than low levels of arousal (e.g., Arsenio & Lover, 1997).

Of the 52 studies included, only six studies found a non-significant relationship between positive emotion and AB, and no studies found a negative relationship between positive emotion and AB. Four studies found a non-significant relationship between positive emotion and AB (Arsenio & Ramos-Marcuse, 2014; Erreygers et al., 2017; Ortiz Barón et al., 2018; Roos et al., 2011), and two studies found no significant differences between antisocial and comparison groups (Blair, 1997; van Dijk et al., 2017). These authors cited individual differences (e.g., age, gender, emotionality, aggression levels) and contextual factors (e.g., type of witness to their AB, victim reactions) as possible explanations for their non-significant findings. Others suggested that AB and depression often present together (Wolff & Ollendick, 2006) which may have dampened positive affect, leading to the null findings. Conflicting findings further highlight the importance of considering the complex interplay between individual child characteristics and contextual factors on the relationship between positive emotion and AB.

It is important to note that while findings indicated that the experience of positive emotion in relation to AB was common in community, clinical and forensic samples, many children reported only experiencing negative emotions in relation to AB (e.g., Arsenio & Lover, 1997; Benenson et al., 2008; Callender et al., 2010). For those who anticipated experiencing positive emotion, it explained only part of their emotional motivation, as some children engaged in AB to reduce negative emotions such as boredom (e.g., Owens et al., 2007), or due to a sense of injustice (e.g., Vylegzhanina et al., 2017) or feelings of threat (e.g., Gabaldón, 2021). Other children also reported mixed negative and positive emotions (e.g., anger and excitement) in relation to AB (e.g., Gabaldón, 2021). These findings are consistent with QVT, which suggests that violence can be motivated by either positive or negative emotion (Howard, 2011). Although these theories were developed to focus on the motivations for violence in adults, our findings suggest that it can be extended to the role of emotion in relation to childhood aggression. In contrast to the adult literature, most studies focused on positive emotion in relation to proactive rather than reactive aggression. Most studies that investigated proactive and reactive aggression suggested that children may experience positive emotion in relation to both forms of aggression (de Castro et al., 2005; Gasser et al., 2012; Owens, et al., 2000, 2005) and appear to differentiate between them, judging proactive aggression as more deserving of negative consequences and viewing retaliatory aggression as less harmful (Gasser et al., 2012). However, Arsenio et al. (2004) found that reactive aggression was not related to positive emotion once proactive aggression was accounted for, whereas proactive aggression was related to positive emotion when controlling for reactive aggression. This supports QVT’s distinction between the two forms of aggression in relation to emotion motivation, but suggests that positive emotion may not play a strong role in motivating children whose aggression was a response to provocation. Due to the small number of studies investigating positive and negative emotions in relation to both forms of aggression, further research is needed to clarify if positive emotion is specific to child proactive aggression. Furthermore, no studies in the current review examined children’s appraisal of negative emotional experience as positive (e.g., anger or fear subjectively experienced as positive), in relation to their AB with the exception of Panayiotou et al. (2015), who found that bullying was related to greater joy in response to anger imagery. Disentangling the role of the positive appraisal of emotions irrespective of their valence as highlighted in adult theories is an important area for future research in children and adolescents.

Many studies failed to explicitly investigate the timing of positive emotion in relation to antisocial acts. When the timing of affect was made explicit, studies typically focused on only one stage of Chester’s (2017) proposed feedback loop. Most studies examined positive affect in anticipation of, or during the antisocial act, with fewer studies examining positive emotion following AB. Research on revenge in adults has indicated that its affective consequences are often the opposite to their expectations, with many left feeling worse afterwards (Carlsmith et al., 2008). Thus the experience of positive affect may not be constant across all three stages of the antisocial act, and the impact of changes in affective valence on later AB is unclear. Future research should aim to investigate whether the influence of positive affect on AB differs depending on its valence, timing, and intensity across the different stages of the proposed feedback loop (Chester, 2017). Furthermore, most studies in the current review were cross-sectional, making it difficult to determine the direction of the relationship between positive emotion and AB over time. However, the findings of longitudinal studies examining reciprocal relationships between these two constructs suggest that positive emotion drives AB, as opposed to vice versa (Byck et al., 2015; Dishion et al., 1996; Harden et al., 2012; Wong et al., 2023). Future research examining the stability of positive affect in the context of AB in relation to its severity and persistence would help to inform the optimal timing of prevention and intervention.

Child individual characteristics including age, gender, and temperament/personality traits were identified as potential moderators of the positive emotion—AB link. Evidence relating to the happy victimizer effect indicated that this phenomenon is more common, but not exclusive to, younger children, with its decline with increasing child age attributed to improved emotion perspective taking skills (Helwig, 2007; Malti & Krettenauer, 2013). While both boys and girls experienced positive emotion in relation to AB, this appeared to be more common in boys, particularly in peer contexts (e.g., Aluja-Fabregat & Torrubia-Beltri, 1998; Benenson et al., 2008; Ching et al., 2014; Roos et al., 2011). Boys are more physically aggressive (Lansford et al., 2012), display higher levels of CU traits and more externalising symptoms than girls (Allen et al., 2020), and are more vulnerable to unhealthy risk-taking in peer contexts, especially when under the influence of alcohol and other substances (McMurran et al., 2010). There are gender differences in the general expression of emotion, with boys showing more externalizing emotions (e.g., anger) than girls, and girls showing greater happiness and internalizing emotions (e.g., sadness, sympathy; Chaplin & Aldao, 2013). Parents may also show different responses to emotions of boys and girls, with fathers paying more attention to anger and ‘disharmonious happiness’ in boys and internalizing emotions in girls (Chaplin et al., 2005). Similar to adult theory and research, positive affect was linked to temperament/personality traits, most notably CU/psychopathic traits (Feilhauer et al., 2013; Hawes et al., 2019; Kunimatsu et al., 2012; Spidel et al., 2011), sensation-seeking (Aluja-Fabregat & Torrubia-Beltri, 1998; Byck et al., 2015; Harden et al., 2012), and dominance (Nassem & Harris, 2015; Mishna et al., 2010). These traits tend to be higher in boys (Else-Quest et al., 2006; Essau et al., 2006), thus findings indicating that positive affect linked to antisocial acts was more prevalent in boys may be due to a complex interplay between individual differences including child age, gender, temperament, and socialization processes (e.g., parent and peer influences, cultural values and attitudes towards gender roles and emotion expression).

Studies exploring contextual influences chiefly focused on gang membership and affiliation with delinquent peers. Weenink (2014) found evidence for solidarity excitement within groups who planned, participated in, and discussed antisocial acts together. These youth laughed about their acts of aggression, and experienced positive affect as part of strengthening their bonds with their peers. Similar findings were present in community and clinical samples, with delinquent and mixed dyads enjoying conversations about deviant topics (Dishion et al., 1996). Peer modelling and reinforcement of positive emotion expressed in relation to AB provides a possible explanation for the increased likelihood of AB in groups (Warr, 2002). Other peer influences on AB were also identified such as a desire to impress others, to gain peer acceptance and peer pressure (Owens et al., 2000; 2005). The effect of delinquent peer affiliation appears to vary across gender, with males being more strongly impacted than females (Piquero et al., 2005). However, most studies of peer influence on positive emotion featured predominantly male samples (e.g., Dishion et al., 1996; Weenink, 2014), with few studies including female delinquents or gang members. None of the eligible, identified studies explored parental influences on positive emotion-related AB, highlighting this as an area in great need of attention in future research.

Current review findings align with those of a previous systematic review and meta-analysis (Malti & Krettenauer, 2013), confirming the presence of the happy victimiser phenomenon in community, forensic and clinical samples. Children and adolescents understood that real and hypothetical acts of AB were immoral but did not consistently attribute moral emotions to these transgressions because they anticipated they would experience, or actually experienced positive emotion during the transgression (e.g., excitement, joy, amusement). Interestingly, one study (Cimbora & McIntosh, 2003) found that youth with CO-CD (but not youth with AO-CD) attributed significantly more excitement and happiness to the transgressor. The authors suggested that early caregiver experiences may explain the group difference, as caregivers are well-known influences on negative emotions related to the development of conscience, such as guilt (Eisenberg, 2000). Childhood-onset CD is associated with difficult child temperament, poor parenting practices, poor quality parent–child relationships and child maltreatment, while those with adolescent-onset CD tend to have experienced better quality parenting and parent–child relationships in childhood (Hawes & Allen, 2016). As such parents of children with child-onset CD may have displayed positive affect in relation to their own AB, with the antisocial act either directly experienced or witnessed by the child, explaining why children with child-onset CD attributed more excitement and happiness to the transgressor. Similarly, parents may show greater encouragement of the child’s expression of AB-related positive affect, particularly fathers in relation to their sons (e.g., Chaplin et al., 2005). While current theories emphasize the influence of parenting on negative, externalizing or conscience-related emotions (e.g., Eisenberg et al., 1998; Kochanska, 1993), these theories should consider extending their frameworks to encompass positive emotion experienced in the context of AB to promote further research.

Current review findings should be interpreted with reference to the quality of included studies. Measures of positive emotion were varied and many measures were not standardised, with many studies relying on unpublished interviews. As such, these measures of positive emotions may have low reliability and validity. Most studies relied on a single informant and used questionnaires or interviews to assess one or both constructs of interest, which are subjective in nature and open to mood, memory and social desirability biases. Future research should adopt a multi-informant approach, to ensure that children’s emotions and behavior are assessed from different perspectives in a variety of contexts, and make greater use of objective methods such as behavior observation and experimental tasks. Due to the diversity of study designs, samples, informants and measures used, it is difficult to make direct comparisons between studies, and precludes the use of meta-analysis to quantify the strength of the association and the influence of potential moderators. Several studies did not report the age range or proportion of boys and girls in their sample, or effect sizes, and missing data and attrition rates (longitudinal studies) were not always clearly described. Forty percent of the studies had at least one ‘can’t tell’ rating indicating that many did not include the required information for an accurate quality assessment. Researchers should include more detailed information to facilitate the transparency and reproducibility of their research, and to enable the influence of research quality to be formally examined via meta-analysis. However, most of the included studies (81%) met at least 60% of the quality criteria, increasing our confidence in the validity of review findings. Finally, all but four studies (Cao et al., 2023; Hasegawa 2023; Vylegzhanina et al., 2017; Wong & McBride, 2018) were conducted in Western nations, limiting the generalizability of our findings. Further research in non-Western countries is needed given cultural differences in AB, emotion expression and social and moral norms. The current review did not identify any studies that addressed the positive subjective experience of emotions typically classified as negative such as fear and anger, although it is possible that our search terms did not lead to their detection given the focus on emotions typically classified as positive. Thus it is important that future reviews and individual studies on the positive emotion—AB link in children and adolescents consider the subjective appraisal of ‘negative’ emotions as positive regardless of valence in future research.

This review also possesses considerable strengths. To our knowledge, this is the first review that provides an extensive overview of the relationship between positive emotion and AB in children and adolescents. The deliberate incorporation of diverse methodologies, designs and methods, coupled with the use of narrative synthesis, facilitates the integration of findings and captures the individual child-level and contextual factors that influence this phenomenon. Furthermore, the diversity of samples suggests that our findings can be generalised to boys and girls from early childhood to late adolescence in community, forensic, and clinical populations. This review included studies from 16 different nations across five continents, suggesting that the experience of positive affect in relation to AB may be universal for children and youth.

## Conclusions

The current review systematically explored and synthesised findings from 52 studies, with findings suggesting that positive emotion experienced in relation to a variety of antisocial acts appears to contribute to their initiation and maintenance, consistent with adult theories highlighting the role of positive affect in aggression (e.g., Chester, 2017; Howard, 2011). This result was largely consistent across different study designs, samples, informants and methods. However, positive affect-related AB appears to be more pronounced for boys, children higher in CU traits, dominance, sensation-seeking, and in the presence of deviant peers. Our findings suggest that experiencing positive emotion in relation to AB is a common in youth, regardless of their level of engagement with AB. Therefore this phenomena should not be pathologized, but rather considered in relation to the child’s developmental stage, temperament, and the interpersonal contexts that the AB arises in. Our suggest that current theories of moral development and AB in children and adolescents should incorporate positive affect into their frameworks to guide future research. This will aid in the development of prevention and treatment strategies addressing the affective motivations underlying AB in childhood and adolescence. Prevention and early intervention programs could offer psychoeducation about emotions and emotion regulation strategies from the preschool years onwards, to support children to use healthy, prosocial strategies to experience positive emotion or to alleviate negative mood. Community programs for high-risk children (e.g., those who have experienced maltreatment or live in a deprived neighbourhood) and juvenile justice systems may wish to consider offering activities that provide a sense of enjoyment and excitement, as prevention or intervention measures for youth who may otherwise seek thrills from engaging in unhealthy risk-taking or AB.

## Supplementary Information

Below is the link to the electronic supplementary material.

Supplementary file1 (DOCX 18 KB)

## Data Availability

Data will be made available on request.
