# Supplementary material for: When Being Bad Feels Good: A Systematic Review of the Relationship Between Positive Emotion and Antisocial Behavior in Children and Adolescents
Source: Clin Child Fam Psychol Rev. 2024 Jul 3;27(3):832–62. doi: 10.1007/s10567-024-00493-4 (PMC11486775; doi:10.1007/s10567-024-00493-4)
Supplement: Supplementary file 1 — Supplementary file1 (DOCX 18 KB) [file 10567_2024_493_MOESM1_ESM.docx]

## **Appendix A: Supplementary Information**

**Table A.1**

*Free text search terms (Titles, Abstracts, Key Words)*

| **Antisocial behaviour** |
| --- |
| “antisocial behaviour” OR  “antisocial behavior” OR  “anti social behaviour” OR  “anti social behavior” OR  “oppositional defiant disorder” OR  “antisocial peers” OR  “antisocial peer group*” OR  “anti social peers” OR  “anti social peer group*” OR  “antisocial friend*” OR  “anti social friend*” OR  “deviant peer*” OR  “deviant peer group*” OR  “deviant friend*” OR  “conduct disorder” OR  “conduct problems” OR  “conduct problem” OR  “behaviour problem” OR  “behavior problem” OR  “behaviour problems” OR  “behavior problems” OR  “problem behaviour*” OR  “problem behavior*” OR  “disruptive behaviour” OR  “disruptive behavior” OR  “illegal act” OR  “illegal acts” OR  Crime OR  Criminal OR  Illegal OR  Bullies OR  Bully* OR  Cyberbully* OR  Cyberbullies OR  delinquen* OR  aggressi* OR  violen* OR  vandal* OR  theft OR  thiev* OR  steal* OR |
| **Positive emotion** |
| “positive emotion*” OR  “positive emotions” OR  “positive state*” OR  “positive affect” OR  “positive mood” OR  “positive moods” OR  “positive feeling” OR  “positive feelings” OR  pleasur* OR  excite* OR  joy* OR  amuse* OR  delight* OR  happy OR  happiness OR  pride* OR  proud OR  joke* OR  humour* OR  laugh* OR  funny OR |
| **Truncation* |

**Table A2: Index terms for each electronic database**

| **Database** | **Antisocial behaviour index terms** | **Positive emotion index terms** | **Limits** |
| --- | --- | --- | --- |
| APA PsychNET    (APA thesaurus of psychological index terms) | “antisocial behavior” OR “oppositional defiant disorder” OR “conduct disorder” OR “behavior problems” OR “disruptive behavior disorders” OR “juvenile delinquency” OR “crime” OR “relational aggression” OR “bullying” OR “cyberbullying” OR “violent crime” OR “vandalism” OR “theft” | “positive emotions” OR “pleasure” OR “happiness” OR “pride” OR “jokes” OR “laughter” | Humans AND  (Child OR  adolescent) N  NO books |
| Embase  (EMTREE term, unexploded) | “antisocial behavior” OR “oppositional defiant disorder” OR “oppositional behavior” OR “conduct problem” OR “conduct disorder” OR “problem behavior” OR “disruptive behavior” OR “disruptive behavior disorder” OR “bullying” OR “cyberbullying” OR “crime” OR “criminal behavior” OR “juvenile delinquency” OR “delinquency” OR “delinquent” OR “aggression” OR “violent crime” OR “violent behavior” OR “violence” OR “theft” OR “vandalism” | “positive emotion” OR “positive affect” OR “pleasure” OR “excite” OR “excitement” OR “joy” OR “amusement” OR “happy” OR “happiness” or “pride” OR “humor” OR “laughter” | Child OR  adolescent |
| International Bibliography of the Social Sciences  (ProQuest Thesaurus) | “antisocial behavior” OR “delinquency” OR “deviance” OR “juvenile delinquents” OR “juvenile delinquency” OR “crime” OR “criminal behavior” OR “aggression (behavior)” OR “vandalism” OR “theft” OR “stealing” OR “bullying” OR “cyberbullying” OR “violence in schools” OR “violence” OR “violent crime” | “joy” OR “happiness” OR “jokes” OR “humour” | English Language |
| Scopus | N/A- only used free text terms | N/A- only used free text terms | English Language |
| PubMed | N/A – only used free text terms  “antisocial behaviour” OR  “antisocial behavior” OR  “anti social behaviour” OR  “anti social behavior” OR  “oppositional defiant disorder” OR  “antisocial peers” OR  “antisocial peer group*” OR  “anti social peers” OR  “anti social peer group*” OR  “antisocial friend*” OR  “anti social friend*” OR  “deviant peer*” OR  “deviant peer group*” OR  “deviant friend*” OR  “conduct disorder” OR  “conduct problems” OR  “conduct problem” OR  “behaviour problem” OR  “behavior problem” OR  “behaviour problems” OR  “behavior problems” OR  “problem behaviour*” OR  “problem behavior*” OR  “disruptive behaviour” OR  “disruptive behavior” OR  “illegal act” OR  “illegal acts” OR  Crime OR  Criminal OR  Illegal OR  Bullies OR  Bully* OR  Cyberbully* OR  Cyberbullies OR  delinquen* OR  aggressi* OR  violen* OR  vandal* OR  theft OR  thiev* OR  steal* OR  “attention deficit and disruptive behavior disorders” OR  “disruptive, impulse control, and conduct disorders” OR  “criminal behaviour” OR  “juvenile delinquency” | N/A – only used free text terms  “positive emotion*” OR  “positive emotions” OR  “positive state*” OR  “positive affect” OR  “positive mood” OR  “positive moods” OR  “positive feeling” OR  “positive feelings” OR  pleasur* OR  excite* OR  joy* OR  amuse* OR  delight* OR  happy OR  happiness OR  pride* OR  proud OR  joke* OR  humour* OR  laugh* OR  funny OR  “emotions” OR  “expressed emotion” OR  “smiling” | Humans AND English language AND  (Child OR  adolescent) |
